# Phase-controlled photon blockade in optomechanical systems

**DOI:** 10.1016/j.fmre.2022.07.009

**Published:** 2022-07-30

**Authors:** Yong-Pan Gao, Cong Cao, Peng-Fei Lu, Chuan Wang

**Affiliations:** a School of Electronic Engineering and the State Key Laboratory of Information Photonics and Optical Communications, Beijing University of Posts and Telecommunications, Beijing 100876, China; b School of Artificial Intelligence, Beijing Normal University, Beijing 100875, China

**Keywords:** Photon blockade, Optomechanics, State phase, Correlation function, Broad frequency range

## Abstract

The manipulation of photons is a key technology for obtaining optical quantum information. In this study, we present a phase-modulated optomechanical system comprising two coupled cavity resonators and illustrate the phase-controlled photon blockade in the system. The coupling phase of the cavities reveals the interference of photons and introduces an unconventional photon-blockade effect. We also study the influence of the energy level fineness on the photon blockade and resonant frequency of the mechanical mode. Numerical simulations demonstrate that photon blockade can occur over a wide range of system parameters. These results have several implications for understanding the role of the state phase in quantum cavity optomechanics and provide a promising method for the realization of optomechanical quantum devices using photon blockade.

## Graphical abstract

**Figure:**
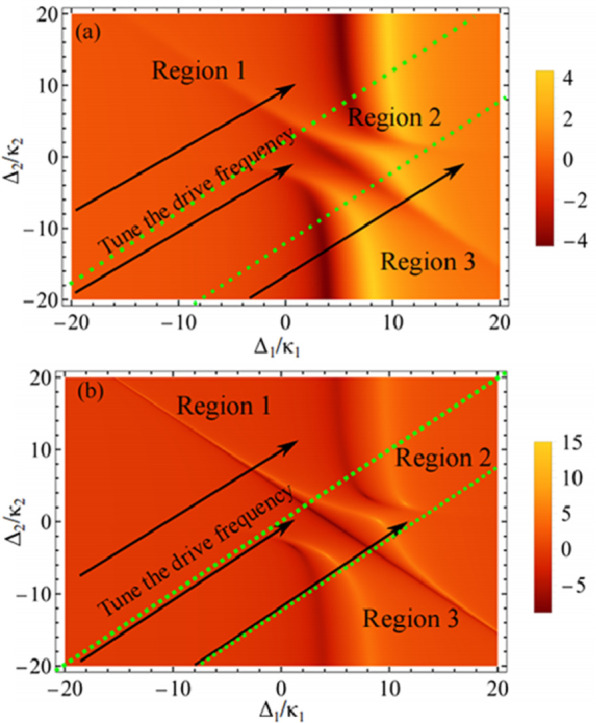

## 1 Introduction

Optomechanics describes the interaction between optical and mechanical fields through the radiation pressure of light [1], [2], [3], [4] that provides a promising platform for light manipulation and optical quantum information processing. Generally, in an optomechanical system, optical and mechanical modes both exist and interact with each other, and the modes can cover the frequency range of the terahertz (THz) and megahertz (MHz) bands, respectively. Thus, it provides an efficient link between optical communication, microwave photonics, and quantum information science.

During the past decades, optomechanics has attracted extensive research interest in terms of both classical properties and quantum dynamics. Specifically, in the field of classical properties, optomechanical systems are employed to achieve the optomechanically induced transparency [5], [6], [7], [8], [9], optical frequency combs [10], [11], [12], solitons [13], [14], [15], [16], and chaotic communication [17], [18], [19]. In the context of quantum dynamics, optomechanical systems are widely used for the modulation of photon distribution [20], [21], [22], [23], entangled photon generation [24], [25], and photon blockade [26], [27], [28], [29].

Here, the concept of photon blockade refers to the phenomenon wherein photons pass through an optical passage [26], [30], [31] and the passage of the first photon blocks the transmission of the second photon. There are two different explanations for this photon blockade phenomenon: the conventional photon blockade [30], [32], [33], [34] refers to the process wherein when a photon passes through an optical structure, the energy level of the structure is excited, which tends to be non-uniform; thus, the second photon cannot pass through. The unconventional photon blockade [31], [35] refers to the process in which photons pass through the structure, quantum interference occurs when destructive interference occurs, and photon blockade can appear. The photon blockade has been realized in cavity-atom systems [30]; meanwhile, it has been widely studied in various optical systems such as optomechanical systems [36], [37], and the optomagnonical systems [32].

Photon blockades are highly significant in optical quantum logic and photon detection devices. Therefore, they have an important effect on quantum information processing and quantum computation. Photon blockade in the optomechanical system was first studied in 2011 by Rabl [26] and Girvin [38]. Later, it was widely studied in various optomechanical structures, including non-reciprocal systems [28], parity-time-symmetric cavity systems [33], [39], and high-order coupling optomechanics [27], [40].

State phase refers to the phase difference between two superposed orthogonal quantum states. The state phase is an extremely important parameter for both unconventional photon blockade and fundamental quantum mechanics. Previous studies on optomechanics have focused on unconventional photon blockades under an unchangeable coupling phase. Specifically, the construction of a tunable state phase is discussed in the optomechanical photon blockade schemes, and the analytical solution of quantum states in a complex parameter space is difficult to express. Motivated by this, we intend to study the performance of the coupling phase in an optomechanical system during a photon blockade.

In this study, we investigate the properties of photon blockade of a phase-coupled photonic dimer comprising two whispering gallery mode optomechanical resonators and present a general solution for the coefficients of the quantum state. By introducing a tunable coupled structure, we introduce a relatively nonreciprocal phase [41], [42], [43] between the two coupled resonators that can be used to tune photon blockade and multiphoton excitation. We believe that the current research has potential theoretical guidance for understanding and utilizing state phases that could further achieve high-precision quantum metrology and quantum telecommunication [44], [45].

## 2 Phase-coupled cavity optomechanical system

**Fig. 1:**
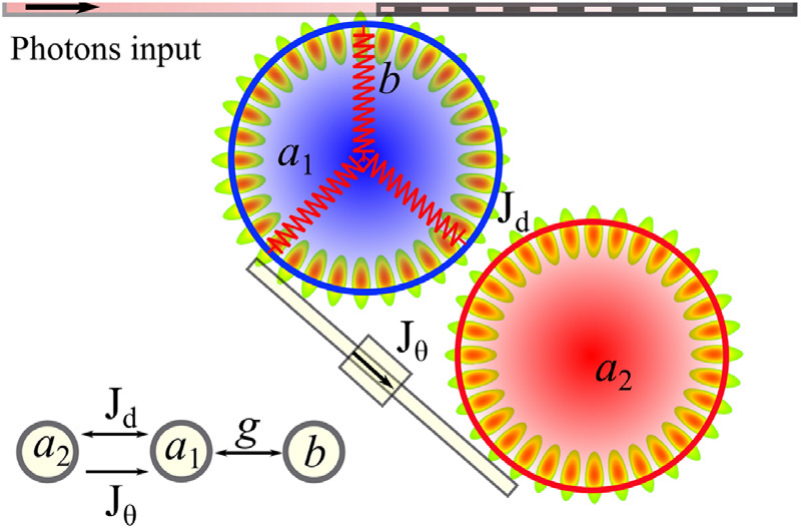
**(Color online) The scheme of the phase-coupled cavity optomechanical system.** The system is driven by coherent sources through the fiber tapper. There are two types of coupling dynamics in the system: direct coupling $J_{d}$ and single direction waveguide coupling $J_{\theta }$. The optical mode in the first cavity is marked with $a_{1}$, which is coupled with the mechanical mode b under the coupling strength g. The optical mode of the cavity-2 is denoted by $a_{2}$.

To explore the dynamic behavior of the phase-coupled optomechanical system, the model comprises two coupled whispering gallery mode(WGM) optomechanical resonators, as shown in Fig. 1. One optomechanical WGM cavity was excited using a fiber trapper. The optomechanical resonator supports both optical mode $a_{1}$ and mechanical mode b under coupling strength g. The optomechanical cavity was also coupled to another WGM resonator $a_{2}$. The coupling between these two WGM cavities contains two parts: the direct coupling strength $J_{d}$ and the one-way waveguide-induced complex coupling part $J_{\theta }$. Subsequently, there is a coupling phase θ that can be tuned by the coupling fiber length or phase modulators.

The Hamiltonian of the phase-coupled dual-cavity optomechanical system is described as:

(1) $$\begin{array}{ccc} H & = & \omega_{1}a_{1}^{†}a_{1}+\omega_{2}a_{2}^{†}a_{2}+\omega_{m}b^{†}b+J_{1}a_{1}^{†}a_{2}+J_{2}a_{1}a_{2}^{†}-ga_{1}^{†}a_{1}\left(b^{†}+b\right)\\ & & +\;(Ea_{1}^{†}e^{-i\omega_{l}t}+E^{*}a_{1}e^{i\omega_{l}t}) \end{array}$$

where $\omega_{1}$ and $\omega_{2}$ are the eigenfrequencies of the cavity-1 ($a_{1}$) and cavity-2 ($a_{2}$), respectively. $\omega_{m}$ denotes the mechanical resonance frequency.

The driven strength of the system is expressed as $E=\sqrt{2\kappa_{1}P/\hbar \omega_{1}}$, where P is the driven power of the system and $\kappa_{1}$ denotes the decay rate of cavity-1. Here, we set the coupling parameters to $J_{1}=J_{d}$ and $J_{2}=J_{d}+J_{\theta }$. $J_{1}$ should be real because it originates from the direct coupling of $J_{d}$, whereas $J_{2}$ is complex because it contains the phase-coupling term $J_{\theta }$, which is the coupling phase of the system. In the rotating frame reference to the drive frequency $\omega_{l}$, the Hamiltonian becomes:

(2) $$\begin{array}{ccc} H & = & \Delta_{1}a_{1}^{†}a_{1}+\Delta_{2}a_{2}^{†}a_{2}+\omega_{m}b^{†}b+J_{1}a_{1}^{†}a_{2}+J_{2}a_{1}a_{2}^{†}-ga_{1}^{†}a_{1}\left(b^{†}+b\right)\\ & & +\;(Ea_{1}^{†}+E^{*}a_{1}) \end{array}$$

Here, $\Delta_{1}=\omega_{1}-\omega_{l}$ and $\Delta_{2}=\omega_{2}-\omega_{l}$ correspond to the detuning between each cavity and drive field, respectively. When the system is in large detunings, it can be simplified using the adiabatic elimination of the mechanical mode [46]. To diagonalize the nonlinear interaction terms in all frequency regions and study the system from the perspective of super modes, we used the polaron transform given by $V=\exp[ga_{1}^{†}a_{1}\left(b^{†}-b\right)/\omega_{m}]$ [26], under the assumption that ($g\ll \omega_{m}$), the transformed system can be written as $H_{1}=V^{†}HV$.

(3) $$\begin{array}{ccc} H_{1} & = & \Delta_{1}a_{1}^{†}a_{1}+\Delta_{2}a_{2}^{†}a_{2}+\omega_{m}b^{†}b+J_{1}a_{1}^{†}a_{2}+J_{2}a_{1}a_{2}^{†}-\frac{g^{2}}{\omega_{m}}{\left(a_{1}^{†}a_{1}\right)}^{2}\\ & & +\;(Ea_{1}^{†}+E^{*}a_{1}) \end{array}$$

When focusing on the transformed Hamiltonian, the optical and mechanical parts are separated, and $\frac{g^{2}}{\omega_{m}}{\left(a_{1}^{†}a_{1}\right)}^{2}$ is the same as that of the Kerr Hamiltonian [47]. Here, only the optical properties of the system are considered; therefore, we remove the mechanical part of the Hamiltonian as:

(4) $$\begin{array}{ccc} H_{\mathrm{NM}} & = & \Delta_{1}a_{1}^{†}a_{1}+\Delta_{2}a_{2}^{†}a_{2}+J_{1}a_{1}^{†}a_{2}+J_{2}a_{1}a_{2}^{†}-\frac{g^{2}}{\omega_{m}}{\left(a_{1}^{†}a_{1}\right)}^{2}\\ & & +\;(Ea_{1}^{†}+E^{*}a_{1}) \end{array}$$

Although the mechanical part of the Hamiltonian is deleted, it also plays an important role in the dynamic behavior. In fact, the frequency of the mechanical part determines the strength of optical nonlinearity that is the origin of photon blockade.

In practice, the optical cavity is usually an open dissipate system, and the dissipation term must be introduced into the Hamiltonian and imaginary parts into its eigenfrequency as:

(5) $$\begin{array}{ccc} H_{\mathrm{NM}} & = & \left(\Delta_{1}-i\kappa_{1}\right)a_{1}^{†}a_{1}+\left(\Delta_{2}-i\kappa_{2}\right)a_{2}^{†}a_{2}+J_{1}a_{1}^{†}a_{2}+J_{2}a_{1}a_{2}^{†}-\frac{g^{2}}{\omega_{m}}{\left(a_{1}^{†}a_{1}\right)}^{2}\\ & & +\;(Ea_{1}^{†}+E^{*}a_{1}) \end{array}$$

Therefore, we should discuss the influence of thermal noise, assuming that the system works at room temperature; subsequently, the thermal photons are near zero according to the thermal photon number $n_{t}h=1/\left(e^{\hbar \omega /k_{B}T}-1\right)$. Thermal phonons cannot be ignored; however, the role of thermal phonons is to cause changes in the optical frequency but have no effect on the overall properties of the system. In the actual process, we must only define the optical eigenfrequency of the cavity under the influence of thermal phonons as the optical eigenfrequency of the cavity. The state of the system can be represented by the photon number states |m,n〉 of the two cavities, that is: $|\Psi \left(t\right)\rangle =\sum_{\begin{array}{c} m=1\\ n=1 \end{array}}^{\infty }C_{m,n}\left(t\right)|m,n\rangle$

By substituting this into the Schrödinger equation, $\frac{i\hbar \partial }{\partial t}|\Psi \left(t\right)\rangle =H_{NM}|\Psi \left(t\right)\rangle$, we obtain:

(6) $$\begin{array}{ccc} i{\dot{C}}_{m,n}\left(t\right) & = & \left[m\left(\Delta_{1}-i\kappa_{1}\right)-\frac{m^{2}g^{2}}{\omega_{m}}+n\left(\Delta_{2}-i\kappa_{2}\right)\right]C_{m,n}\left(t\right)+\sqrt{m\left(n+1\right)}J_{1}C_{m-1,n+1}\\ & & +\;\sqrt{\left(m+1\right)n}J_{2}C_{m+1,n-1}+\sqrt{m}EC_{m-1,n}+\sqrt{m+1}E^{*}C_{m+1,n} \end{array}$$

In this equation, we have $C_{i,j}=0$ when i,j<0. For a dissipative system, the probability that the system is in the lower energy state is higher under weak driving, as $C_{m,n}\;\gg \;C_{k,l}$ when m+n<l+k, and $C_{0,0}\approx 1$ under weak driving ($E\ll \kappa_{1,2}$) conditions. Then, the evolution equation of $C_{m,n}$ can be solved as:

(7) $$\begin{array}{ccc} i{\dot{C}}_{m,n}\left(t\right) & = & \left[m\left(\Delta_{1}-i\kappa_{1}\right)-\frac{m^{2}g^{2}}{\omega_{m}}+n\left(\Delta_{2}-i\kappa_{2}\right)\right]C_{m,n}\left(t\right)+\sqrt{m\left(n+1\right)}J_{1}C_{m-1,n+1}\\ & & +\;\sqrt{\left(m+1\right)n}J_{2}C_{m+1,n-1}+\sqrt{m}EC_{m-1,n} \end{array}$$

To clearly demonstrate the evolution of the system, and considering that the system is under weak driving and dissipation, the total number of particles in the system is approximately conserved, and the total number of photons and photon number state of the second cavity are employed to represent the state of the system. In this expression, we can directly replace the state |m,n〉 with |N,n〉 as:

(8) $$\begin{array}{ccc} i{\dot{C}}_{N,n}\left(t\right) & = & \left[m\left(\Delta_{1}-i\kappa_{1}\right)-\frac{m^{2}g^{2}}{\omega_{m}}+n\left(\Delta_{2}-i\kappa_{2}\right)\right]C_{N,n}\left(t\right)+\sqrt{m\left(n+1\right)}J_{1}C_{N,n+1}\left(t\right)\\ & & +\;\sqrt{\left(m+1\right)n}J_{2}C_{N,n-1}\left(t\right)+\sqrt{m}EC_{N-1,n} \end{array}$$

Under the steady-state condition, each coefficient $C_{N,n}$ remains unchanged with time, denoting $\frac{dC_{N,n}}{dt}=0$. The problem of solving differential equations becomes a problem of solving simultaneous equations as follows:

(9) $$\begin{array}{ccc} 0 & = & \left[m\left(\Delta_{1}-i\kappa_{1}\right)-\frac{m^{2}g^{2}}{\omega_{m}}+n\left(\Delta_{2}-i\kappa_{2}\right)\right]C_{N,n}\left(t\right)\\ & & +\;\sqrt{m\left(n+1\right)}J_{1}C_{N,n+1}\left(t\right)+\sqrt{\left(m+1\right)n}J_{2}C_{N,n-1}\left(t\right)+\sqrt{m}EC_{N-1,n} \end{array}$$

The general solution of the above equation is difficult to solve directly; therefore, we turn to its recurrence. The matrix form of the equations can be expressed as follows:

(10) $$M_{N}{\vec{C}}_{N}=S_{N}{\vec{C}}_{N-1}$$

Here, matrix $M_{N}$ is an N+1-order matrix. To be consistent with the number of photons, we mark the rows and columns of the matrix from number 0, and the matrix element can be expressed as $M_{N}\left(i,i\right)=i\left(\Delta_{1}-i\kappa_{1}\right)-\frac{i^{2}g^{2}}{\omega_{m}}+\left(N-i\right)\left(\Delta_{2}-i\kappa_{2}\right)$, $M_{N}\left(j,j-1\right)=\sqrt{\left(N-j)(j+1\right)}J_{1}$ and $M_{N}\left(j,j+1\right)=\sqrt{(N-j+1)j}J_{2}$, and all other elements are 0. The jth element of ${\vec{C}}_{N}$ can be denoted as ${\vec{C}}_{N}\left(j\right)=C_{N,j-1}$. Matrix $S_{N}$ contains matrix elements expressed as $S_{N}\left(i,i\right)=\sqrt{N-i}$. Therefore, the general term of the N-photon state is:

(11) $${\vec{C}}_{N}=\left(\prod_{1}^{N}M_{N}^{-1}S_{N}\right){\vec{C}}_{0}$$

The determinants and matrices above are infinite-dimensional, however, most of the elements are 0. In fact, for simplicity, we must only consider all quantities to ${\vec{C}}_{N}$ with an N-dimensional matrix and vectors. This equation shows that the state of the system is divided by the total number of photons, which also corresponds to the approximately conserved total number of photons.

To investigate the photon blockade of the system, we specifically demonstrate the properties of the system under two-photon conditions. The expressions for higher-dimensional cases are complex and will not be discussed here. For the two-photon state, the coefficients can be solved as:

(12a) $$C_{01}=\frac{4\omega_{m}EJ_{2}}{\omega_{m}A+2g^{2}\left(2\Delta_{2}-i\kappa_{2}\right)}$$

(12b) $$C_{10}=\frac{2\omega_{m}E\left(2\Delta_{2}-i\kappa_{2}\right)}{\omega_{m}A+2g^{2}\left(2\Delta_{2}-i\kappa_{2}\right)}$$

(12c) $$C_{11}=8E^{2}J_{2}\left(2\Delta_{2}-i\kappa_{2}\right)\omega_{m}^{2}P_{1}/Q$$

(12d) $$C_{20}=2\sqrt{2}E^{2}{\left(2\Delta_{2}-i\kappa_{2}\right)}^{2}\omega_{m}^{2}P_{2}/Q$$

(12e) $$C_{02}=8\sqrt{2}E^{2}J_{2}^{2}\omega_{m}^{2}P_{1}/Q$$

where the parameters area being as follows:

(13a) $$P_{1}=-4g^{2}+\left(2\Delta_{1}+2\Delta_{2}-i\kappa_{1}-i\kappa_{2}\right)\omega_{m}$$

(13b) $$P_{2}=-2g^{2}+\left(2\Delta_{1}+2\Delta_{2}-i\kappa_{1}-i\kappa_{2}\right)\omega_{m}$$

(13c) $$\begin{array}{ccc} Q & = & \left(2g^{2}\left(2\Delta_{2}-i\kappa_{2}\right)+A\omega_{m}\right)(8g^{4}\left(2\Delta_{2}-i\kappa_{2}\right)+2g^{2}B\omega_{m}\\ & & +\;A\left(2\Delta_{1}+2\Delta_{2}-i\kappa_{1}-i\kappa_{2}\right)\omega_{m}^{2}) \end{array}$$

(13d) $$A=4J_{1}J_{2}-\left(2\Delta_{1}-i\kappa_{1}\right)\left(2\Delta_{2}-i\kappa_{2}\right)$$

(13e) $$B=8J_{1}J_{2}-\left(2\Delta_{2}-i\kappa_{2}\right)\left(3\left(2\Delta_{1}-i\kappa_{1}\right)+2\left(2\Delta_{2}-i\kappa_{2}\right)\right)$$

The photon-blockade effect can be described using a second-order correlation function. For the proposed system, considering the cases where there are two photons in cavity-1 and cavity-2, and cavity-1 and cavity-2 each having one photon is necessary. The correlation functions for the three conditions are expressed as follows:

(14a) $$g_{1}^{\left(2\right)}\left(0\right)=\frac{2{\left|C_{20}\right|}^{2}}{{\left({\left|C_{10}\right|}^{2}+{\left|C_{11}\right|}^{2}+2{\left|C_{20}\right|}^{2}\right)}^{2}}≃\frac{2{\left|C_{20}\right|}^{2}}{{\left|C_{10}\right|}^{4}}$$

(14b) $$g_{2}^{\left(2\right)}\left(0\right)=\frac{2{\left|C_{02}\right|}^{2}}{{\left({\left|C_{01}\right|}^{2}+{\left|C_{11}\right|}^{2}+2{\left|C_{02}\right|}^{2}\right)}^{2}}≃\frac{2{\left|C_{02}\right|}^{2}}{{\left|C_{01}\right|}^{4}}$$

(14c) $$\begin{array}{ccc} g_{12}^{\left(2\right)}\left(0\right) & = & {\left|C_{11}\right|}^{2}/\left[\left({\left|C_{10}\right|}^{2}+{\left|C_{11}\right|}^{2}+2{\left|C_{20}\right|}^{2}\right)\times \left({\left|C_{01}\right|}^{2}+{\left|C_{11}\right|}^{2}+2{\left|C_{02}\right|}^{2}\right)\right]\\ & ≃ & \frac{{\left|C_{11}\right|}^{2}}{{\left|C_{10}\right|}^{2}{\left|C_{01}\right|}^{2}} \end{array}$$

When the correlation function fulfills $g^{2}=0$, the corresponding cavity is in the photon blockade state. When the first cavity works in the perfect photon blockade state, we require the condition $|C_{20}|=0$ and the relation:

(15a) $$\frac{g^{2}}{\omega_{m}}\left(-4\Delta_{2}^{2}+2\kappa_{2}^{2}\right)+4\Delta_{2}^{2}-\Delta_{2}\kappa_{2}\left(2\kappa_{1}+3\kappa_{2}\right)+\Delta_{1}\left(4\Delta_{2}^{2}-\kappa_{2}^{2}\right)=0$$

(15b) $$\frac{2g^{2}}{\omega_{m}}\Delta_{2}\kappa_{2}-8\Delta_{1}\Delta_{2}\kappa_{2}+\kappa_{2}^{2}\left(\kappa_{1}+\kappa_{2}\right)-4\Delta_{2}^{2}\left(\kappa_{1}+3\kappa_{2}\right)=0$$

From the above analysis, we observed that the system can achieve photon blockade under various conditions. For example, when $\Delta_{1}=\Delta_{2}=g^{2}/2\omega$ and $\kappa_{1}=-\kappa_{2}$, it reveals the parity-time symmetry [48], [49] condition, and the system operates in the perfect photon blockade station, which is consistent with previous results [33].

## 3 Phase-induced photon blockade and photon excitation

Owing to the limitations of fabrication technologies at nanoscale, it is difficult for the system to achieve perfect photon blocking. In practice, we only require the second-order correlation function of photons to be lower than a threshold value. Therefore, studying the variances of the correlation function under parameter tuning, combined with the expression of the second-order correlation function of the system, is particularly important.

Some basic parameters of the system were chosen to calculate the correlation function of the system. Here, we use the following parameters in the following text: the damping of the cavity is set as $\kappa_{1}=\kappa_{2}=10$MHz, the mechanical frequency is set as 20 MHz, the direct coupling strength is $J_{1}=5$MHz, and the optomechanical coupling strength is chosen to be 30MHz. For simplicity, we choose the relation of detuning as $\Delta_{1}=\Delta_{2}=\Delta$, and the pumping amplitude of the system is set to 10 kHz.

**Fig. 2:**
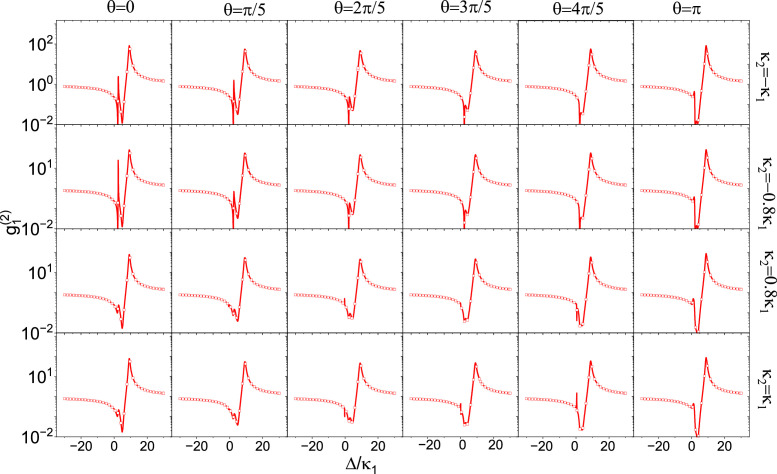
**(Color online) The correlation functions of the system under different coupling phases when the two cavities have the same values of dissipation or gain.** The damping of the cavity is $\kappa_{1}=10$ MHz, the mechanical frequency is 20 MHz, the couplings strength are set as $J_{1}=5$ MHz and $J_{2}=50e^{i\theta }$ MHz, and the optomechanical coupling strength is 30 MHz.

To study the influence of the coupling strength, we set the coupling parameter to $J_{2}=25e^{i\theta }$ MHz. We also show the relation between the correlation function $g_{1}^{2}\left(0\right)$ and the detuning Δ with different damping rates of cavity-2 and different coupling phases θ, and the results are shown in Fig. 2. We can clearly observe that the influence of the state phase on the photon-blocking region g<0.1 and the photon bunching region g>10 is particularly obvious. When $\kappa_{2}=-\kappa_{1}$, the system operates in the PT-symmetric state, and the minimum value of the correlation function increases as the phase increases. This indicates that the photon-blocking effect weakens with an increase in the relative phase. Similarly, we observe the same trend in profile change when $\kappa_{2}=-0.8\kappa_{1}$. However, when the parameter $\kappa_{2}$ is positive, as shown in rows $\kappa_{2}=0.8\kappa_{1}$ and $\kappa_{2}=\kappa_{1}$, the slope of the change is the opposite. Photon blockade becomes stronger when the coupling phase changes from zero to π. This opposite condition arises from the interference of the complex frequency Δ+iκ and the complex coupling $J_{2}$. When cavity-2 is a gain cavity, the interference brought by the 0” coupling phase is the strongest, which reveals the largest photon-blocking effect. By contrast, if cavity-2 is a loss cavity, the phase of the complex frequency is reversed. When the coupling phase is π, the interference is the strongest, indicating the largest photon-blocking effect.

In addition, compared to the positive detuning parts of the system, the influence of the phase appears mainly on the blue sideband detuning part. This is because mechanical coupling increases the energy levels of the system, which modulates the photon excitation regions, and the system works in the conventional photon blockade mode. However, the photons are in the unconventional photon blockade condition in the blue sideband detuning part, and the system properties are modulated by the state phase.

**Fig. 3:**
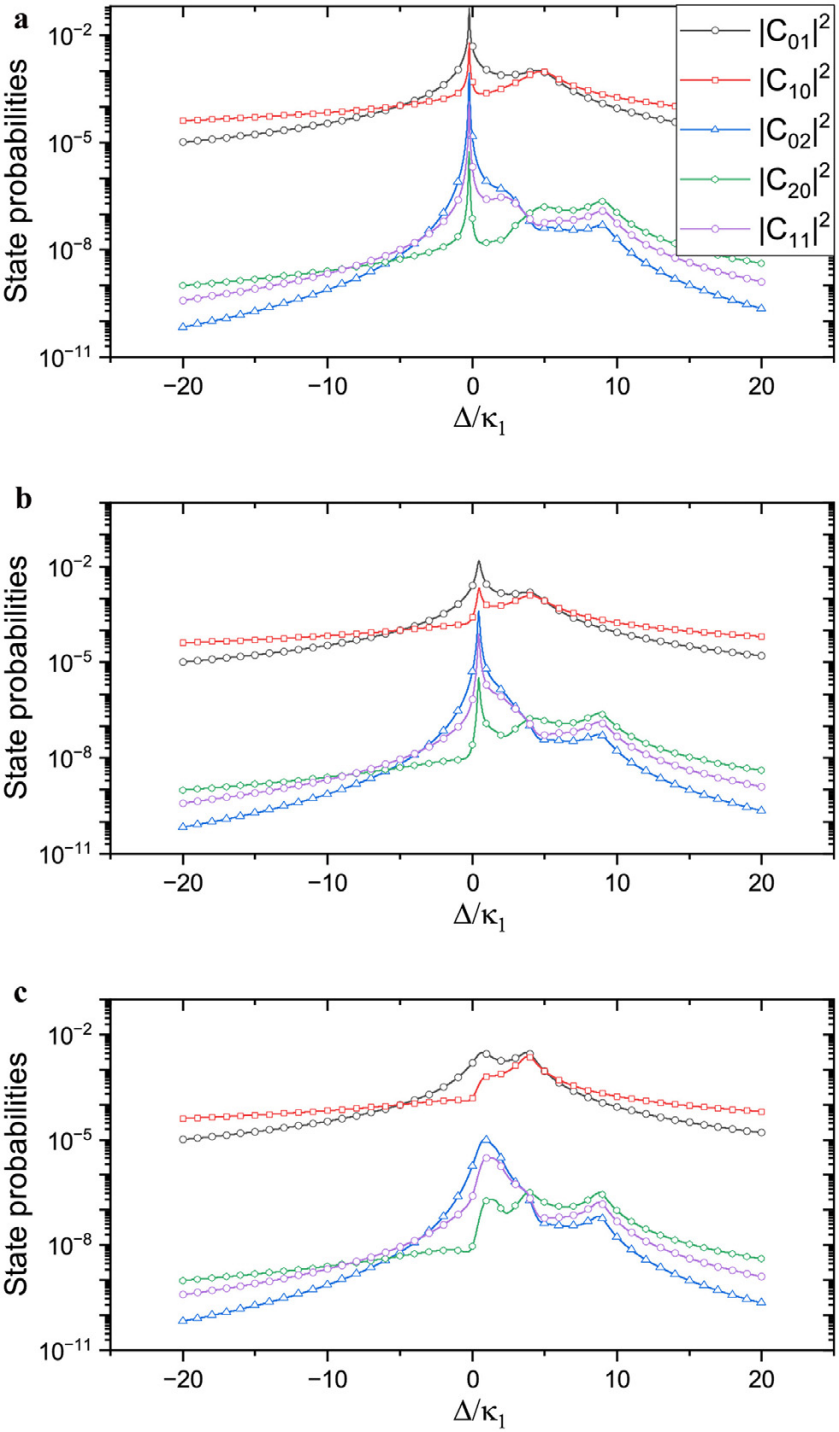
**Photon excitation under different coupling phases.** Here the coupling phase is chosen as 2π/5 in (a), 4π/5 in (b), and π in (c). We set $\kappa_{2}=\kappa_{1}$, other parameters are similar with Fig. 2.

The number of photons in the system is also a key factor in the photon-blockade effect. In the simulation, shown in Fig. 3, we set the coupling phase θ as 2π/5, 4π/5, and π, respectively. The damping rate of the cavity was $\kappa_{2}=\kappa_{1}$. We observe that the photon excitation is considerably different at different phases: when the coupling phase is 2π/5, the probability of the nonzero photon state is greatly enhanced when the pump detuning is near 0. However, the probability of the number of photons is considerably lower than 1. When the coupling phase is 4π/5, we observe that there is a certain probability of enhancement. However, the maximal value of the probability is suppressed. When the coupling phase approaches π, the maximum probability continues to be suppressed. Overall, the coupling phase demonstrated a significant effect on the photon excitation process of the system. Furthermore, we observed that all lines in the system exhibit the same peak position. This is because the relative phases of the different states are not involved, and the resonances are approximately the same for different particle numbers. They are all n times the cavity eigen frequency, so the resonance peaks in the figure are almost at the same position.

**Fig. 4:**
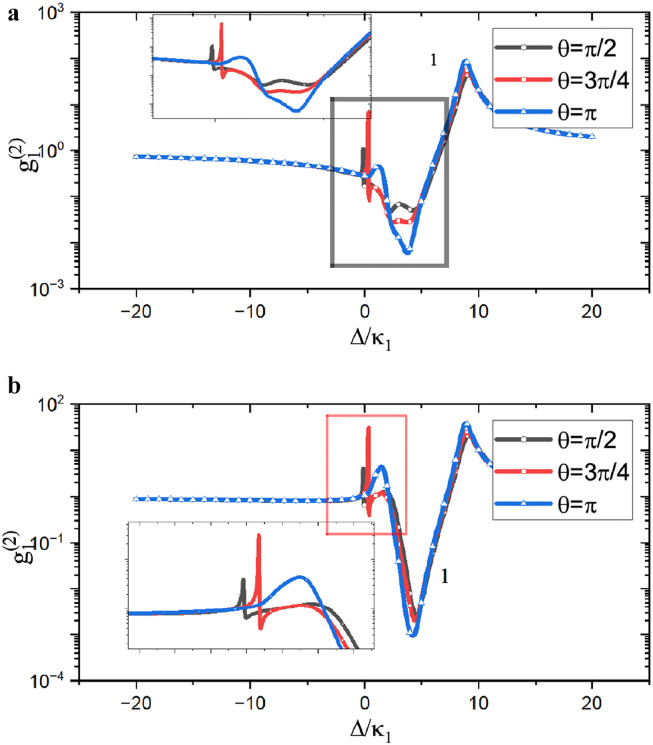
**Correlation functions of the two resonators vary with the pumping frequency.** (a) The correlation function of cavity-1. (b) The correlation function of cavity-2. The parameters are chosen similar with Fig. 2.

As dynamical behavior is discussed in a coupled resonator system, studying the photon blockade phenomenon in each cavity resonator is important. Here, we show the correlation functions for cavity-1 and cavity-2 in Fig. 4a and b, respectively. We observed that the phase term has almost the same influence on the photon blockade in the blue sideband detuning region. However, when the system works in the red sideband detuning region, the influence of the state phase is different. Similarly, the resonance point of the energy levels is located at the red sideband detuning position for the optomechanical cavity, so the resonant points are slightly different for the two cavities. Subsequently, the state phase completely dominates the properties of the system at the blue sideband detuned position. Therefore, the relation between the second-order correlation function and phase of the two cavities is consistent in the blue sideband detuning region. For the red sideband detuning part, the correlation function of the system is no longer affected by the phase owing to the influence of the energy level resonance; therefore, the correlation function is inconsistent with the phase change. When comparing the influence of the coupling phase, we find that the peak of the π phase coupling is flat because the interface is destructive under this condition. This is because the correlation function is not significantly enhanced even if the system is in a strongly excited state, and the curve is relatively flat with π phase coupling.

## 4 Phase-controlled photon blockade under different system parameters

**Fig. 5:**
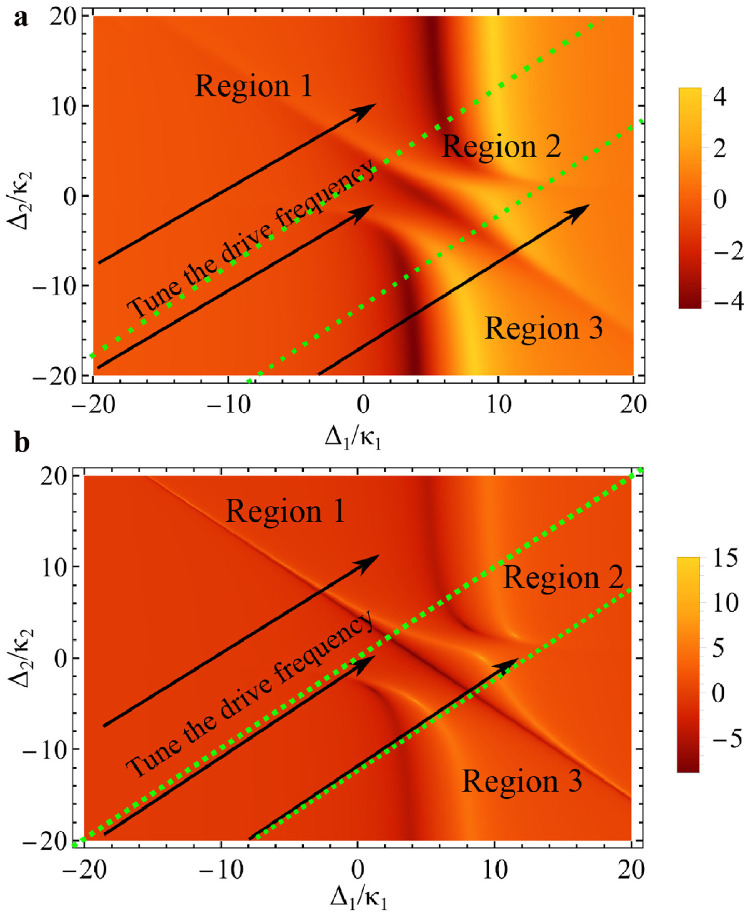
**Correlation function under different cavity detuning.** (a) The dissipative coupled cavities condition. (b) The parity-time-symmetric cavities condition. The parameters are similar with Fig. 2.

We studied the properties of the correlation function in coupled dissipative cavities with decay rate $\kappa_{2}=\kappa_{1}$, as well as in the coupled parity-time-symmetric cavity with dissipation $\kappa_{2}=-\kappa_{1}$ in Fig. 5. The coupling phase between resonators was set to π/2. We observe that the two conditions demonstrate extremely similar variation trends with the change in detuning, but the correlation functions of the two cavities exhibit extremely large order-of-magnitude differences. As observed in the parity-time-symmetric system, there are exceptional points due to the balanced distribution between dissipation and gain, and the system includes fully degenerate eigenvalues at exceptional points, and sensitivity to perturbations [50]. The transformation of the system properties at this point could be enhanced, therefore, the correlation order of magnitude for the parity-time-symmetric state is much stronger.

In Fig. 5, the system changes along with the arrow direction with the detuning. Referring to the direction of the arrow and the changing trend of the graph in the direction of the arrow, the system is divided into three regions using the green dotted line. To ensure the presence of unconventional photon blocking, it is necessary for the system to operate in the second region, where there are multiple peaks in the correlation function. Region 2 represents the condition wherein the cavities of the systems are in the near-resonant condition, which can be explained as follows. When the frequency difference between the two cavities is considerable, the coupling between the cavities is weakened, for which the properties of the cavity will no longer be influenced by the resonance phase. Comparing these two figures, the requirements for the resonance point are different when the system is in different dissipationgain states. The state of parity-time-symmetric resonators is not conducive to the generation of a photon blockade when the system is in a strict resonance state.

**Fig. 6:**
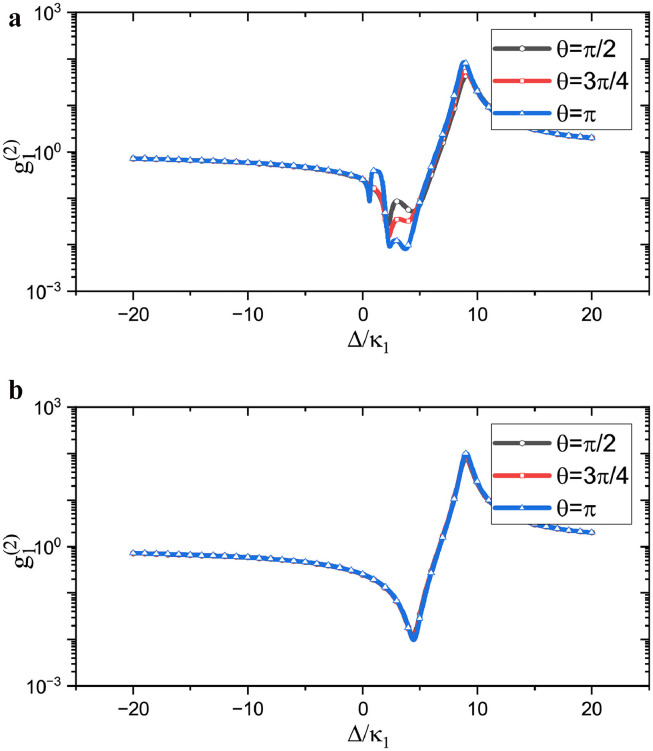
**Influence of the cavity decay rate on photon blockade.** (a) The low-decay rate condition with $\kappa_{2}=0.01\kappa_{1}$. (b) The high-decay rate condition with $\kappa_{2}=100\kappa_{1}$. Other parameters are the same as in Fig. 2.

In addition, the magnitude of dissipation can influence the occurrence of photon blockade. Therefore, the low-dissipation cavity with $\kappa_{2}=0.01\kappa_{1}$ and the high-dissipation cavity with $\kappa_{2}=100\kappa_{1}$ conditions are discussed and shown in Fig. 6a and b, respectively. Obviously, the variation of the correlation shows more characteristics in the resonant region in high-quality factor cavities, whereas in the high-dissipation cavities, the change in the correlation function with phase can be ignored. This is because the bandwidth of the energy levels is expanded, which reduces the resolution of the frequency domain of interference between the two cavities. Therefore, a resonator with a high fineness level plays an important role in realizing the sensitive phase control of photon blocking.

**Fig. 7:**
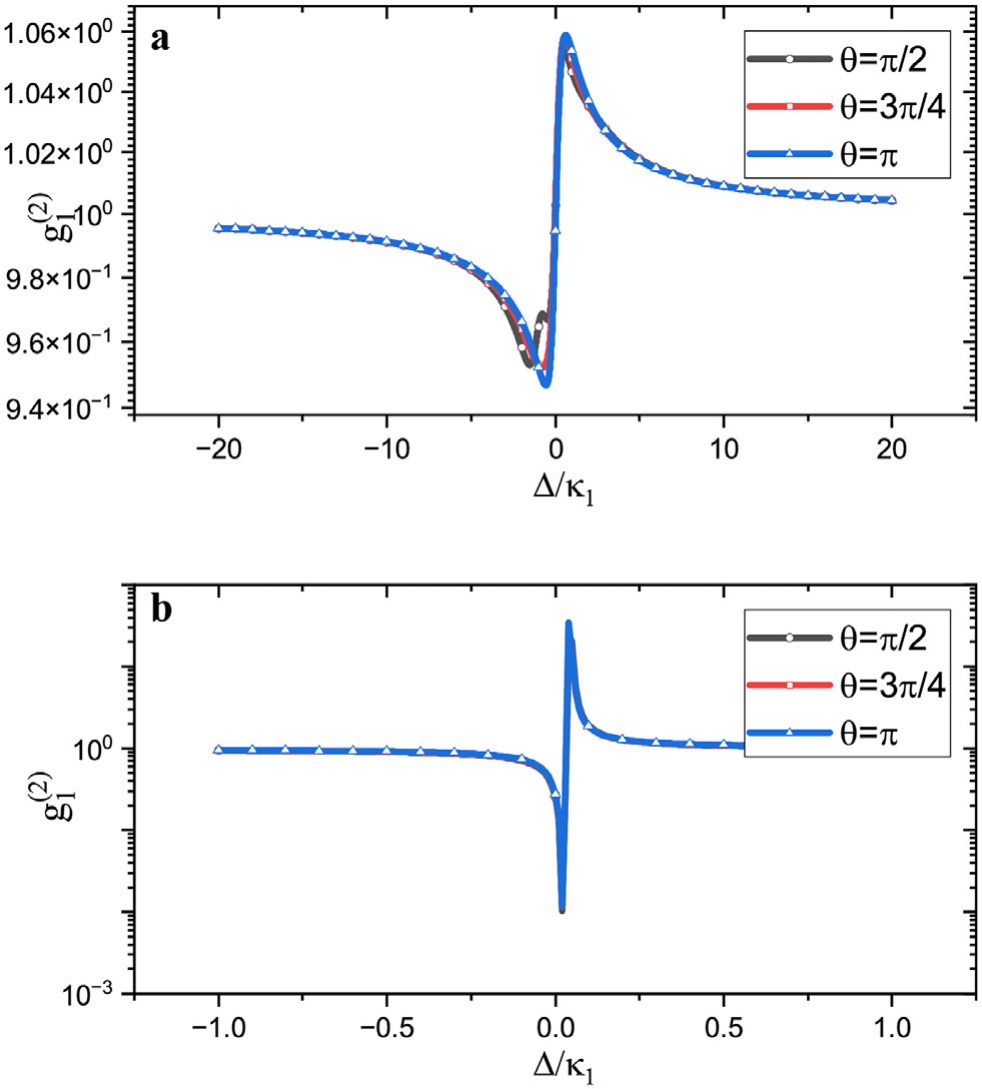
**Photon blockade under the high frequency mechanical resonator.** The mechanical resonator has the frequency $\omega_{m}=200\kappa_{1}$. (a) shows the two passive cavities condition. (b) shows the parity-time-symmetric cavities condition. Other parameters are the same as in Fig. 2.

Moreover, the frequency of the mechanical mode in optomechanical systems can be tuned using state-of-the-art technologies. We show a high-frequency mechanical resonator with resonant frequency $\omega_{m}=200\kappa_{1}$ and damping rate $\kappa_{2}=\kappa_{1}$ in Fig. 7a and gain $\kappa_{1}=-\kappa_{2}$ in Fig. 7b, respectively. We observe that although the coupling phase can considerably change the correlation function, as shown in Fig. 7a, the photon correlation function is always close to one with no photon blocking occurs. However, the correlation function no longer varies with the coupling phase in the parity-time-symmetric system, as shown in Fig. 7b. As the correlation function can be much smaller than one, the system can still be used to achieve photon blockade without modulation by the coupling phase. The above numerical results demonstrate the importance of the mechanical mode in an optomechanical system. The generation of photon blockade is not only influenced by the optical parameters but also the frequency of the mechanical mode affects the occurrence of photon blockade, especially the occurrence of photon blockade under the action of the state phase.

## 5 Conclusion

We presented a phase-coupled resonator system and studied the effects of the coupling phase on the photon blockade effect. Through a numerical study, we observed an enhancement in the photon blockade by tuning the coupling phase. In addition, by considering different dissipative resonators in the system, we observed different behaviors of photon blockade with the effect of the phases. When the system is completely parity-time-symmetric, the zero phase causes the largest photon blockade. When all cavities are dissipative, we set the coupling phase as π to obtain the maximal photon blockade. Furthermore, the low dissipative cavities can increase the phase controllability of the photon blockade effect, as the reduction in interference resolution is avoided. Finally, we discuss the effect of the mechanical mode on the photon blockade performance. The system has a higher equivalent Kerr coefficient at low mechanical frequencies, for which it is easier to achieve photon blockade. Compared with previous photon blockade schemes, our phase control schemes are more practical because the optical phase modulation schemes are mature and precise. Our study has several important implications for understanding the role of the state phase and realization of photon blockade. In addition, it will benefit the study of quantum information processing and optical qubit manipulations.

## Declaration of competing interest

The authors declare that they have no conflicts of interest in this work.
